# SARS-CoV-2 IgG Seroprevalence among Blood Donors as a Monitor of the COVID-19 Epidemic, Brazil

**DOI:** 10.3201/eid2804.211961

**Published:** 2022-04

**Authors:** Daniel Gonçalves Chaves, Ricardo Hiroshi Caldeira Takahashi, Felipe Campelo, Maria Clara Fernandes da Silva Malta, Isabelle Rocha de Oliveira, Edel Figueiredo Barbosa-Stancioli, Maísa Aparecida Ribeiro, Marina Lobato Martins

**Affiliations:** Fundação Hemominas, Belo Horizonte, Brazil (D.G. Chaves, M.C.F. da Silva Malta, M.A. Ribeiro, M.L. Martins);; Universidade Federal de Minas Gerais, Belo Horizonte (R.H.C. Takahashi, F. Campelo, I.R. de Oliveira, E.F. Barbosa-Stancioli);; Aston University, Birmingham, UK (F. Campelo)

**Keywords:** COVID-19, respiratory infections, severe acute respiratory syndrome coronavirus 2, SARS-CoV-2, SARS, coronavirus disease, zoonoses, viruses, coronavirus, blood donor, epidemic model, seroprevalence, Brazil

## Abstract

During epidemics, data from different sources can provide information on varying aspects of the epidemic process. Serology-based epidemiologic surveys could be used to compose a consistent epidemic scenario. We assessed the seroprevalence of severe acute respiratory syndrome coronavirus 2 (SARS-CoV-2) IgG in serum samples collected from 7,837 blood donors in 7 cities of Brazil during March–December 2020. Based on our results, we propose a modification in a compartmental model that uses reported number of SARS-CoV-2 cases and serology results from blood donors as inputs and delivers estimates of hidden variables, such as daily values of SARS-CoV-2 transmission rates and cumulative incidence rate of reported and unreported SARS-CoV-2 cases. We concluded that the information about cumulative incidence of a disease in a city’s population can be obtained by testing serum samples collected from blood donors. Our proposed method also can be extended to surveillance of other infectious diseases.

Coronavirus disease (COVID-19), caused by severe acute respiratory syndrome coronavirus 2 (SARS-CoV-2), emerged in China in 2019 and spread around the world in 2020. By August 2021, >200 million persons were infected by SARS-CoV-2 and >4 million had died (*1*). A large proportion of infected persons remain asymptomatic or have only mild symptoms (*2*,*3*). The role of all infected persons should be considered in the maintenance of disease transmission, especially because asymptomatic or mildly symptomatic persons often are not tested or reported to public health authorities (*4*). In addition, because of economic, political, and social difficulties, molecular tests for detecting SARS-CoV-2 often are limited, particularly in developing countries (*5*,*6*). In this context, alternative measures must be explored to generate reliable data that enable government decisions to contain viral spread.

Several studies report a rapid immune response that culminates in the production of SARS-CoV-2 antibodies in the first weeks after infection (*5*–*8*). Assessment of serologic SARS-CoV-2 IgG can be an essential tool to measure the dynamics of virus transmission.

Some authors hypothesized that serosurveillance in blood donors can help monitor the evolution of SARS-CoV-2 infections (*9*–*11*). We conducted a large longitudinal study using the records of reported COVID-19 cases and SARS-CoV-2 serology results from blood donors as inputs into a modified susceptible-exposed-infected-recovered (SEIR) epidemic model (*12*). The proposed model delivers daily estimates of relevant variables that usually stay hidden, including the transmission rate and the cumulative number of reported and unreported cases of infection. We considered the continuous change of the transmission rate, making the estimated variables compatible with the shifting conditions of disease transmission. We used the monthly estimates of cumulative incidence provided by serologic analysis of blood samples to estimate the proportion of reported and unreported cases. The purpose of the model is to constitute a platform for integrating and interpreting of information coming from different sources.

Our study also provides evidence supporting the possibility of using serology from blood samples collected in blood donation centers to estimate the accumulated incidence of disease. Our results cover blood samples collected in 7 blood donation centers. Our proposed method provides a consistent picture of the evolution of COVID-19 in the 7 representative cities and describes the cumulative incidence, daily transmission rate, and proportion of reported and unreported cases.

## Materials and Methods

### Study Population

We enrolled blood donors at 7 blood donation centers (Fundação HEMOMINAS) in Minas Gerais, Brazil, during March 1–December 31, 2020. These 7 blood donation centers account for ≈60% of the blood collections performed by HEMOMINAS and are in the cities of Belo Horizonte, Governador Valadares, Juiz de Fora, Montes Claros, Pouso Alegre, Uberaba, and Uberlândia. We used Epitools (Ausvet, https://epitools.ausvet.com.au) to calculate the number of samples to include monthly from each selected blood center based on the prevalence of COVID-19 cases reported by the municipal health secretaries in each city. However, we analyzed more samples than the Epitools-defined quantity in all centers during all periods. The study was approved by the Ethics Committee of Fundação HEMOMINAS (approval no. CAAE 31087720.2.0000.5118). Data from all donors were collected from the records of each blood donation center.

### Sample Collection and Testing

We randomly selected samples collected for serologic screening (n = 7,854) in the 7 blood donation centers, aliquoted, and froze at −80°C until SARS-CoV-2 IgG testing was performed. We used the SARS-CoV-2 IgG Kit (Abbott, https://www.abbott.com), following the manufacturer’s instructions, to determine the IgG of the nucleocapsid protein of samples that tested positive or negative for SARS-CoV-2. Among the samples tested, we excluded 17 (0.2%) from the study because they corresponded to different donations from the same donors that were IgG positive for SARS-CoV-2. Testing results in different donations from the same donor (n = 17) always showed the same result. We only kept the first donation from each SARS-CoV-2 IgG–positive repeat donor in the analysis.

### Epidemic Model

Studies using dynamic models of COVID-19 epidemics usually use compartmental models with SEIR structures (*13*). We propose a SEIR model that uses the same compartments defined by R. Li et al. (*12*), in which persons are susceptible, exposed (those in the latent period of infection), reported as infected (those that can propagate the virus and are reported in public health statistics), unreported infected, and removed (persons who have either recovered and become immune, at least temporarily, or have died). In addition to the number of persons in each compartment, our model also defines the transmission rate, β, as a variable that changes with time, and differential equations describe the time evolution of variables (Appendix). Our version of the model does not yet consider vaccination or the possibility of reinfection. Vaccination could be included in our model by moving vaccinated persons to the removed compartment, but further studies on the loss of immunity in both recovered and vaccinated persons are needed to elucidate usefulness of the model for longer timeframes. Other issues that could be studied include the response of serology tests in detecting vaccine antibodies and the effect of IgG waning in test results. 

Our model has 2 parameters that are mainly biologically determined: the average time a person stays in the compartment of exposed before changing to infected and the average duration of infection. Other parameters depend on both biologic and social factors.

Most studies related to the dynamic modeling of COVID-19 epidemics consider either a constant or a piecewise constant, β, that changes as governments enact or remove social distancing and other containment measures (*14*). However, the actual dynamics of COVID-19 epidemics vary faster due to the shifting response of populations to virus containment measures. In this study, we assumed that β has a daily value, which we estimated by minimizing the error between the number of reported infected persons delivered by the model and the number of infected persons reported by public health services. This assumption creates an implicit feedback loop that forces the model’s internal variables to adapt to mirror the corresponding real hidden variables of the epidemic process. A model capable of delivering estimates of hidden internal variables of a system is called a state observer (*13*,*15*).

After β is estimated, the remaining model parameter to be found is the fraction (α) of infected persons detected by testing and becoming reported cases. This value is determined by comparing the accumulated number of reported cases with the accumulated incidence indicated by seroprevalence in blood donors.

We assessed our proposed method by using data of apparent COVID-19 lethality (deaths divided by reported cases). We were able to determine apparent lethality because the testing policy used in the state of Minas Gerais defined patients’ COVID-19 testing eligibility on the basis of severity of symptoms. These criteria were very restrictive at first and were relaxed after the testing infrastructure was expanded in July 2020. Therefore, α changed from one fixed value to another fixed value, leading to a change in the apparent lethality by the same factor of the change in α. Because the data relative to deaths, reported cases, and incidence in blood donor samples are mutually independent, our proposed model could be assessed by checking its simultaneous compatibility with these data in all cities. For this purpose, we used the apparent lethality to infer α instead of using the proportion between the accumulated number of reported cases and incidence in blood samples.

### Statistical Analysis

We calculated the number of occurrences of each outcome and the frequencies for categorical variables. We made comparisons by using the Fisher exact test. We calculated the median and interquartile range (IQR) for continuous variables and performed comparisons by using 2-sided Mann-Whitney tests. We calculated all tests and confidence intervals at 95%. We estimated the proportion of positive IgG tests for each blood donation center by aggregating the number of tests and positive results each month after removing repeat donors who had positive tests recorded in previous visits. We used Epitool (Ausvet) to calculate the unadjusted and test-adjusted seroprevalence for sensitivity (90%) and specificity (99%) (*16*,*17*), using Wilson’s CI for apparent prevalence and Blaker’s CI for true prevalence.

## Results

### SARS-CoV-2 IgG Seroprevalence among Blood Donors

**Figure 1:**
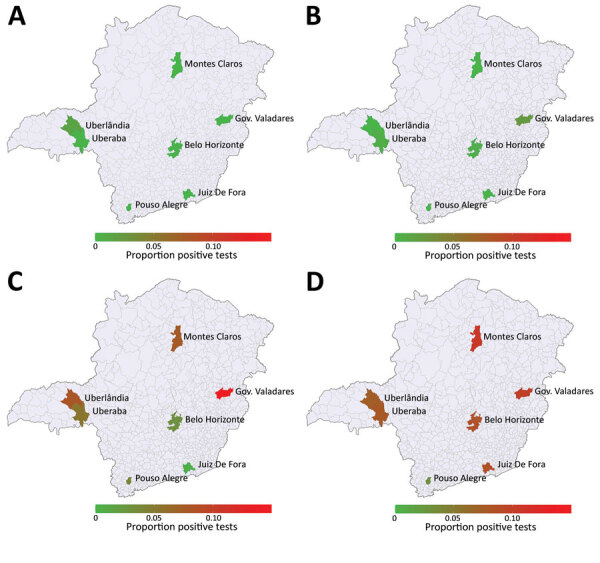
Temporal evolving cumulative severe acute respiratory syndrome coronavirus 2 (SARS-CoV-2) seroprevalence in HEMOMINAS Foundation blood donation centers in 7 cities of Minas Gerais, Brazil, March–December 2020. A) March; B) June; C) September; D) December. Data represent SARS-CoV-2 IgG seropositivity among persons eligible to donate blood. Scale bar represents cumulative proportion of SARS-CoV-2 IgG seropositivity per 100,000 population. Gov., Governador.

Our study included data from 7,837 donors who gave blood at 7 donation centers in Brazil during March 1–December 31, 2020. The total number of samples included in the study represents 6.4% of all blood donations in the selected centers during the study period. Serologic testing to identify SARS-CoV-2 IgG revealed 441 (5.63%) positive blood donors during the study period. When adjusted for sensitivity and specificity of the test, the overall rate of positivity was 5.20% (95% CI 4.65%–5.80%). Male donors had 1.35 (95% CI 1.12–1.63) times the odds of being seropositive than did female donors. The type of donor (first-time vs. repeat donor) did not represent a statistically significant difference between groups who were positive or negative for SARS-CoV-2 IgG. Age also was not a statistically significant difference, either across age groups after adjusting for multiple hypotheses tests using the Holm correction or when regressing the rate of positivity on age by using simple linear regression (Table). We calculated the evolving seroprevalence over all months of 2020 in each blood center and its geographic location in Minas Gerais (Figure 1). In most blood centers, the increase in seroprevalence rates was slower in the first months, accelerated in August, but became faster during October (Appendix Table).

**Table:** Characteristics and severe acute respiratory syndrome coronavirus 2 IgG seroprevalence among blood donors, Brazil, March–December 2020

Characteristics	Total samples	Positive samples	Unadjusted seroprevalence, % (95% CI)	Test-adjusted seroprevalence, % (95% CI)*	Odds ratio (95% CI)
Total	7,837	441	5.6 (5.1–6.2)	5.2 (4.7–5.8)	
Sex					
M	4,284	273	6.4 (5.7–7.1)	6.0 (5.3–6.9)	1.4 (1.1–1.6)
F	3,553	168	4.7 (4.1–5.5)	4.2 (3.5–5.0)	
Age range, y					
16–30	2,895	153	5.3 (4.5–6.2)	4.8 (4.0–5.8)	
31–40	2,330	135	5.8 (4.9–6.8)	5.4 (4.4–6.5)	
41–50	1,701	115	6.8 (5.7–8.1)	6.5 (5.3–7.9)	
51–60	829	32	3.9 (2.8–5.4)	3.2 (2.0–4.9)	
61–70	82	6	7.3 (3.4–15.1)	7.1 (2.7–15.8)	
Donor type					
First-time donor	1,483	72	4.9 (3.9–6.1)	4.3 (3.2–5.7)	0.8 (0.7–1.1)
Repeat donor	6,353	369	5.8 (5.3–6.4)	5.4 (4.8–6.1)	

*Considering sensitivity of 90% and specificity of 99%. Analysis performed by Epitool by using Wilson’s confidence interval for apparent rate of positivity and Blaker’s CI for true rate of positivity.

### Modeling SARS-CoV-2 Infection among the General Population

We used the seroprevalence rates of SARS-CoV-2 IgG among blood donors to infer the proportion of persons in the general population infected in the 7 cities’ blood centers, according to the statistical model established. We chose the α parameter so that the accumulated incidence rate delivered by the model, including the reported and unreported cases, fits the prevalence of COVID-19 in the blood donors in each blood center for each month.

### Model Assessment

**Figure 2:**
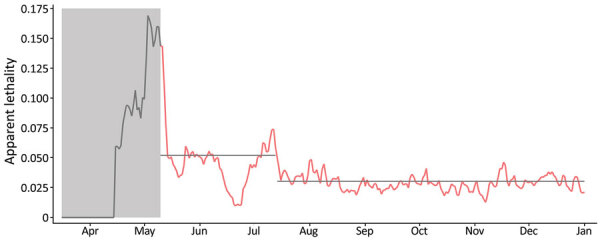
Apparent lethality of coronavirus disease (COVID-19) in 6 cities (Belo Horizonte, Pouso Alegre, Montes Claros, Juiz de Fora, Governador Valadares, and Uberaba), Minas Gerais State, Brazil, April–December 2020. Gray shading and gray data line indicate the beginning of the COVID-19 epidemic in Minas Gerais, days 1–60, in which few cases were reported and the testing infrastructure was still being organized. Red data line indicates days 61–290 of the epidemic. Our model predicted the apparent lethality during days 60–120 to be nearly 5.2% and to fall during days 121–290 to nearly 3.0% (gray horizontal lines). This change corresponds to a nearly 70% increase in the value of α in our model (proportion of infected persons that are reported), assuming that the actual lethality has not changed. We did not include data from the city of Uberlândia in this estimation due to local legislation regulating COVID-19 testing, which resulted in a much larger proportion of people being tested in that municipality than in the other cities. Markings for each month represent the first day of the month.

The evolution of apparent lethality of COVID-19 (deaths divided by reported cases) in the cities that host blood centers (except Uberlândia) suggests that the proportion of infected persons tested changed around day 122, July 16, and increased by nearly 70% (Figure 2). That date coincides with the time when additional laboratories were integrated into the testing infrastructure for SARS-CoV-2 provided by the state government. In the case of Uberlândia, the municipality provided a relevant testing infrastructure in addition to the infrastructure provided by the state government in all other cities. Those data provide independent information about the relative changes in the value of α, which can be used for assessing the model consistency.

We performed this assessment by running the model with α taking the same value in all cities except Uberlândia, assuming a fixed value that we increased by 70% at day 122 (July 16; *t* = 122 days) and that remained fixed on this new value from July 16–December 31. We determined the initial value of α that enabled the best fitting of the observed IgG rates of positivity in the blood centers was 0.18 for the proportion of reported cases up to *t* = 122 days and was 0.31 for *t*>122 days in Belo Horizonte, Governador Valadares, Juiz de Fora, Montes Claros, Pouso Alegre, and Uberaba. For Uberlândia, we found the values were 0.37 for *t*<122 and 0.41 for *t*>122.

**Figure 3:**
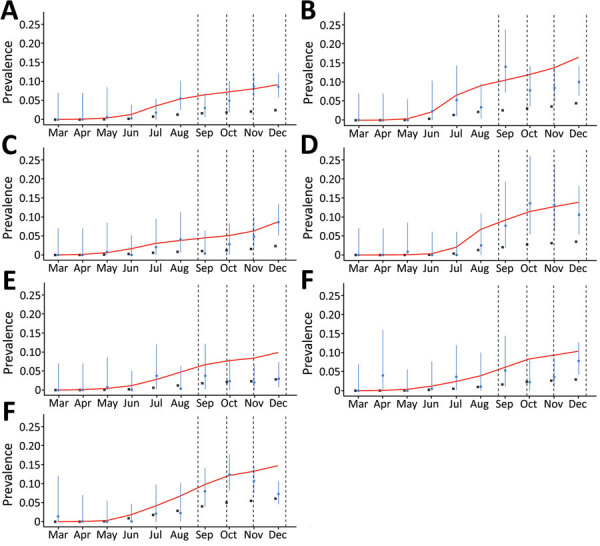
Proportion of severe acute respiratory syndrome coronavirus 2 (SARS-CoV-2) IgG–positive results among blood donors in blood donation centers in 7 cities in Minas Gerais, Brazil, March–December 2020. A) Belo Horizonte; B) Governador Valadares; C) Juiz de Fora; D) Montes Claros; E) Pouso Alegre; F) Uberaba; G) Uberlândia. Blue dots indicate proportion of SARS-CoV-2–positive donors at the end each month; vertical blue lines indicate 95% CIs. Black squares indicate the official cumulative prevalence of reported coronavirus disease cases for each city. Red lines represent model estimates of the number of infected persons, including reported and unreported cases, in each city, as a proportion of the city’s population. Vertical dashed lines indicate national holidays.

We superimposed the seroprevalences in the blood centers each month onto the estimated curves of accumulated number of cases (reported and unreported) as predicted by the model in the respective cities (Figure 3). In 5 cities (Belo Horizonte, Juiz de Fora, Montes Claros, Uberlândia, and Uberaba), these parameter values resulted in reasonable matches with almost all data points. In 2 cities, Governador Valadares and Pouso Alegre, the seroprevalence data in the last period (October–December) were not well-adjusted to the model, suggesting that the pattern of variation of α in those cities could be different from the variations in other cities. However, because Governador Valadares and Pouso Alegre are small cities, a separate analysis of the change in the apparent lethality is not possible, which prevents the possibility of applying the same methodology for refining the estimates when comparing cities of varying populations.

## Discussion

In this study, we evaluated the rate of blood donors who tested positive for SARS-CoV-2 IgG and donated blood in 7 cities in Minas Gerais, Brazil, during March–December 2020. We used the data to estimate the rate of infection in the general population, then used the infection rate in a dynamic model with a SEIR structure.

The higher rate of IgG positivity found in male donors (6.4% vs. 4.7% for female donors) in our study did not agree with the reported COVID-19 cases in Minas Gerais during the same period (49.2% for male vs. 50.8% for female persons). The higher proportion of positive tests among male donors suggests that the epidemiologic profile of infection might change when more persons with asymptomatic or mild COVID-19 are tested, such as expected for blood donors. The rate of positivity associated with sex has been previously observed (*18*), but different works did not identify this association in blood donors (*19*–*21*) or in the general population (*22*).

Concerning differences of positivity between age groups, we found no statistically significant difference in this study. Differences in positivity between age groups is a controversial issue; some studies report higher seroprevalence in younger persons (*19*,*23*), but other studies indicate greater seroprevalence in older persons (*24*) or do not find statistically significant associations between seroprevalence and age (*25*). A study conducted in 133 cities in Brazil found that persons 20–59 years of age, an age group that corresponds to most blood donors included in this study, were more likely to be infected (*26*). The differences between studies might be partly explained by cultural and population issues, making it difficult to consolidate a general conclusion. Loss of statistical power resulting from corrections for testing multiple hypotheses might also play a role in the observed differences not achieving statistical significance, particularly if the effect size is moderate.

Seroprevalence in the blood donation centers showed the proportion of positive donors increased slowly in the first 6 months, and higher proportions of positivity were recorded from August onward, with regional variations. In Minas Gerais, COVID-19 cases increased in June, peaked in August, decreased slowly until October, and then reached the highest numbers in December 2020. Our results agree with this scenario, suggesting that seroprevalence rates in blood donors correlated with reported COVID-19 case rates. A crucial feature of the rate of positivity indicated by serologic testing in the blood centers is that seroprevalence is much greater than prevalence that would be obtained by the accumulated number of reported COVID-19 cases. However, we expected this difference because of underreporting. Notwithstanding, public communication about COVID-19 epidemics is commonly articulated on the basis of reported cases in the community, which strongly underestimates the actual spread of the disease. This difference underscores the convenience of using a model-based approach, as we propose, because it enables the use of measured data for estimating hidden variables, such as the total number of infected persons.

Although all the cities we evaluated had increased COVID-19 positivity rates in December 2020, Governador Valadares had the highest rates. This finding is in consonance with the fact that Governador Valadares had a higher accumulated COVID-19 incidence, 4,227.8 cases/100,000 population, than was seen in Minas Gerais (2,270.1 cases/100,000 population) and Brazil overall (3,383.6 cases/100,000 population). Governador Valadares also had a higher COVID-19 mortality rate, 143 deaths/100,000 population, than Minas Gerais (51.3 deaths/100,000 population) or Brazil (88 deaths/100,000 population).

Several countries are implementing serial SARS-CoV-2 serologic surveillance studies by using blood donors (*19*,*23*,*27*). These studies provide relevant results to complement population seroprevalence data (*19*) and valuable information for decision-making in countries where such data are not available. However, some issues should be considered, including the appropriate test to assess seroprevalence and the threshold for identifying positive and negative samples. Of note, the automated serologic tests that are available were validated by using samples from symptomatic COVID-19 patients with a confirmed diagnosis by reverse transcription PCR (E.W. Eyre et al., unpub. data, https://doi.org/10.1101/2020.07.21.20159038). Results obtained in other studies using the same chemiluminescence test indicated a lower sensitivity to detect SARS-CoV-2 IgG in newly infected persons (*28*), which might affect the extrapolation of seroprevalence data to the population when using blood donors’ samples (*11*,*29*). These data reinforce the need for choosing serologic assays with high sensitivity, specificity, and durable antibody detection, even months after infection (*30*; M. Stone et al., unpub. data, https://doi.org/10.1101/2021.09.04.21262414).

Blood donor–based estimates of SARS-CoV-2 seroprevalence might deviate from the seroprevalence in the general population for several reasons, including the exclusion of populations who cannot donate blood, such as persons <16 and >70 years of age and residents of nursing homes and prisons. The proportion of different groups (e.g., male and female donors, or different age groups) represented in the samples might differ from their respective proportions in population. In addition, recruitment and eligibility criteria for blood donations recommended by the Brazil Ministry of Health during the COVID-19 pandemic excluded asymptomatic candidates who had contact with infected persons <30 days before going to a donation center; those donors had to wait 14 days from the date they were first seen at the blood center before they could donate. The recommendation also excluded potential blood donors who had a COVID-19 diagnosis until >30 days after their symptoms disappeared (*31*). Such guidelines might result in decreased SARS-CoV-2 IgG seropositivity rates among blood donors.

The results of our study comparing prevalence estimates obtained using the SEIR model with actual health system notification data suggest that blood donor serosurveillance data can provide valuable information for monitoring the epidemic and evaluating the effectiveness of measures to fight the virus spread in the cities that have blood donation centers. Our study also showed that the evolution of the epidemic can be considerably different from city to city, even considering cities within the same state in Brazil, suggesting that the application of the proposed SEIR model in other cities would require some strategy of periodic collection of blood samples for serologic analysis from a sufficient number of persons spread across the population.

Some aspects of the proposed modeling approach should be highlighted. First, the procedure for estimating the time-varying transmission rate β for the SEIR model enables a reasonable automatic estimation of that parameter, thus circumventing a major difficulty in COVID-19 modeling (*12*,*14*). As a byproduct, this procedure also eliminates the difficulty usually encountered in determining adequate initial conditions. In fact, the SEIR model, once endowed with the estimation procedure for β, becomes equivalent to a state observer model (*13*,*15*), producing estimates of the model’s hidden variables that will approximate the real unmeasured variables, regardless of initial conditions, provided that the model parameters are reasonable approximations of the actual parameters.

The estimated hidden variables might be quite useful in practice. For instance, β(*t*) provides information that is not contained in the reproduction number, R_t_, because β(*t*) does not vary with the number of recovered persons, representing a better descriptor of social isolation intensity. Perhaps counterintuitively, the cumulative incidence estimate provided by the model can be considered more reliable than the monthly point estimates derived from raw data of serologic analysis in blood centers because the model performs a filtering of the random variation in data that results from sampling.

Concerning assessment of the proposed model, users could choose different values for the α parameter for each city and for each month, according to the outcomes of serologic tests in the respective blood donation centers. Thus, the accumulated incidence of cases estimated by the model would be forced to follow the trajectory of serology results, which would not confirm the model validity. The procedure of model assessment we adopted used the same trajectories for α in 6 cities and got the changes in α from an independent source. The consistency of the model outcomes with the serology results in most of data points, considering cities with rather different trajectories of the epidemics, provides corroboration of our proposed model.

The first limitation of our study is that the stratification of the blood donors by sex or by age would enable the correction of the seroprevalence estimates according to the demographic composition of the general population, leading to more precise results. As we observed (Figure 3), the prevalence in some cities showed a systematic tendency to remain below the values predicted by the model in the last 3 months, which also suggests that a relevant process of seroconversion might exist as IgG wanes. Modeling of such a decay process might help provide the correct interpretation of data in the last months of our experiment. Finally, some of the blood donation centers considered in this study are relatively small (Pouso Alegre and Uberaba), which increases the uncertainty associated with the data collected in those centers, not only by reducing the sample size, but also by reducing the robustness to skewed data.

In conclusion, the results of our study suggest that blood donation centers could be incorporated into COVID-19 surveillance systems with the role of regularly providing quantitative estimates of SARS-CoV-2 seroprevalence in the population. For this purpose, public health agencies should use an epidemic model with a state observer property, which performs a track of some measured variable and produces outputs that converge to the system’s hidden variables. Thus, we propose a specific SEIR epidemic model that performs the adjustment of the transmission rate β such that the model tracks the measured number of reported COVID-19 cases. Our model used seroprevalence data collected in blood centers to adjust the proportion of reported cases considered. This model provided consistent estimates of relevant variables that otherwise would not be accessible, thus supporting a well-informed decision-making process. The methods we propose can be adapted for surveillance of other infectious diseases by using other kinds of input information from sentinel surveillance systems combined with serosurveillance data gathered in blood donation centers.

Appendix Additional information on the use of SARS-CoV-2 IgG seroprevalence as a monitor for the COVID-19 epidemic, Brazil.
