# Supplementary material for: SARS-CoV-2 IgG Seroprevalence among Blood Donors as a Monitor of the COVID-19 Epidemic, Brazil
Source: Emerg Infect Dis. 2022 Apr;28(4):734–42. doi: 10.3201/eid2804.211961 (PMC8962887; doi:10.3201/eid2804.211961)
Supplement: Appendix — Additional information on the use of SARS-CoV-2 IgG seroprevalence as a monitor for the COVID-19 epidemic, Brazil. [file 21-1961-Techapp-s1.pdf]

Article DOI: https://doi.org/10.3201/eid2804.211961

SARS-CoV-2 IgG Seroprevalence among Blood Donors as a Monitor of the COVID-19 Epidemic, Brazil

Appendix

Epidemic Dynamic Model

Most studies that present dynamic models of COVID-19 epidemics use compartmental models with susceptible-exposed-infected-removed (SEIR) structure. This model structure is a variation of the traditional susceptible-infected-removed (SIR) model, with the inclusion of a compartment for Exposed persons, which accounts for the latent period of the infection. A key parameter in those models is the transmission rate, β, which aggregates the effects of some social behaviors in a population such as the mean number of interpersonal contacts of cases, the strength of protection measures in contact situations (for instance, use of facemasks, physical distancing during a contact, and others) and the selective isolation of persons with symptoms, and also the relevant biologic features that determine the ability of the virus to be transmitted when a contact occurs, for instance the mean exhaled viral load, the viral pathogenic mechanisms, and others.

Equation 1

Our study uses a model that follows the SEIR structure:

$$\frac{dS}{dt} = -\frac{\beta}{N} S(I^r + I^n)$$

$$\frac{dE}{dt} = \frac{\beta}{N} S(I^r + I^n) - \frac{1}{Z} E$$

$$\frac{dI^r}{dt} = \frac{\propto}{Z} E - \frac{1}{D} I^r$$

$$\frac{dI^n}{dt} = \frac{(1-\propto)}{Z} E - \frac{1}{D} I^n$$

$$\frac{dR}{dt} = \frac{1}{D}(I^r + I^n)$$

This model is like the one used by Li et al. (*1*). In this model, the compartment $S(t)$ represents the number of susceptible persons in population, $E(t)$ represents the number of exposed persons (the persons that are in the latent period of infection, in which they are not able to propagate the virus yet), $I^r(t)$ represents the number of infected persons (persons who will propagate the virus if they contact a susceptible person) that have been reported in public health statistics, and $I^n(t)$ represents the number of infected persons that have not been reported. The compartment $R(t)$ represents removed persons (persons that have recovered from the disease and consequently have become immune, at least temporarily, or who have died). In this equation, N represents the initial number of persons in the population. In addition, equation 2 performs the computation of the cumulative number of reported infected persons, represented by C^r:

Equation 2

$$\frac{dC^r}{dt} = \propto \frac{E}{Z}$$

Equation 1 has some parameters that are mainly biologically determined. For instance, $Z = 3.69$ (the average time a person stays in the compartment of exposed persons before becoming infected) and $D = 7.0$ (the average duration of infection).

Most published studies concerning the dynamic modeling of COVID-19 epidemics either consider a constant value of β or a piecewise constant value, which changes as social distancing measures are changed by governments. However, the actual dynamics of COVID-19 epidemics varies in a much faster way, due to the varying response of populations to virus containment measures as can be inferred from the growth of infection rates just after holidays or other dates of social events. In addition to β, the α parameter also depends on social factors, representing the fraction of infected persons that are detected by testing and become reported cases.

Thus, for performing a simulation of an actual scenario, estimates for the values of β and α are necessary, as are estimates for the initial values of all model variables, $S(0)$, $E(0)$, $I^r(0)$, $I^n(0)$, $R(0)$. We addressed the issues related to the assignment of values to those parameters and modified the model in equation 1 to transform it to a state observer, as described below. This transformation endowed the model with the capability to auto-adapt to parameter changes while

performing a fitting of the accumulated number of reported cases, C^r, represented in the model, to the corresponding number reported by the public health services.

State Observer for the Epidemic Dynamic Model

State observers are crucial tools that have been developed for monitoring the internal variables of dynamic systems, usually for the purpose of assisting the system control. These tools have many reported applications, mainly in the monitoring and control of complex technological systems, such as in aerospace artifacts, and the chemical industry, among others. Here we offer a general discussion of the idea of state observers. Then, we show the specific state observer that we developed in this study for the monitoring of epidemic processes.

Equation 3

Consider a dynamic system described by the following system of differential equations:

$$\dot{x} = f(x)$$

$$y = g(x)$$

In this system, $f(\cdot)$ represents the system dynamic function, $g(\cdot)$ represents the output measurement function, the vector $x \in R^n$ represents the system internal variables (the system states), and the vector $y \in R^m$ represents the vector of signals that are directly measured on the system. State observers are models that represent dynamic systems that are intended to provide estimates of the system internal signals. We assumed that the exact representation of the system, as described in equation 3, is not available to the analyst.

Equation 4

A state observer for the system in equation 3 can be represented as:

$$\dot{\hat{x}} = f(\hat{x}, \hat{e})$$

$$\hat{y} = \hat{g}(\hat{x})$$

$$\hat{e} = \hat{y} - y$$

In this equation, the functions $\hat{f}(\cdot)$ and $\hat{g}(\cdot)$ are approximated representations of functions $f(\cdot)$ and $g(\cdot)$, $\hat{x}$ represents the vector of estimates of the system internal variables, $\hat{y}$ represents the estimate of output measurement vector, and $\hat{e}$ is the error between the estimated output

vector $\hat{y}$ and the actual measurement vector y. The working principle of the state observers is that the error signal e is fed back into the observer, with this feedback loop designed such that the difference between the system state vector x and the estimate $\hat{x}$ of the state vector provided by the observer converges to zero. After this convergence, the state observer provides estimates of all system signals, including the system internal signals that are not measured directly. The exact convergence can be achieved when $\hat{f} = f$ and $\hat{g} = g$. When the differences between the model ($\hat{f}$, $\hat{g}$) represented in the observer and the actual system dynamics (f,g) are small, the observer state vector, $\hat{x}$, is expected to represent a good estimate of the system internal variables, x.

Equation 5

Most of the state observers that have been studied until recently use an additive feedback of the measurement error, which makes the observer dynamic equation become:

$$\dot{\hat{x}} = \hat{f}(\hat{x}) + K\hat{e}$$

in which $K \in R^{n\times m}$ is a matrix of constant feedback coefficients.

The feedback structure in equation 5 has been used in some published works that propose state observers for SIR-like epidemic models (*2*,*3*). A main drawback of those approaches is that they depend on the function $\hat{f}(\cdot)$ being a reasonable approximation of the function $f(\cdot)$ in the actual system. As we discussed, in the case of COVID-19, the parameter β presents strong and fast variations, which makes the use of those observers difficult because they could be used for very short time horizons in which estimates of β could be considered reasonable approximations of the actual disease transmission rate. In addition, those observers would have no role in the estimation of β values, thus failing to provide the estimate of the variable that would likely be the most crucial.

In our work, we offer a new structure of state observer for SIR-like models in which the infection transmission rate β continuously varies along the timeline. In the proposed technique, the actual accumulated number of COVID-19 cases, C^r, is measured as reported by public health services, and the error between this number and the number $\hat{C}^r$ estimated by the observer is calculated. This error is fed back to the estimator in a rather unusual way. First, we assumed that β is a time-varying parameter, which becomes represented by $\beta(t)$. We run an optimization procedure, searching for a time-varying estimate $\hat{\beta}(t)$ that minimizes that error on each day.

When we find the optimal sequence $\hat{\beta}^*(t)$, the estimates of the other system internal variables appear as byproducts of the optimization procedure that result from the simulation of the model with optimal values of the transmission rate. More specifically, the following cost function is defined in Equation 6 as:

$$J(\hat{\beta}, k) = \sum_{i=k-d}^{k+d} (\log(C^r(i)) - \log(\hat{C}^r(i, \hat{\beta})))^2$$

in which $C^r(i)$ represents the accumulated number of actual reported cases in the city on day *i* and $\hat{C}^r(i,\hat{\beta})$ represents the accumulated number of reported cases calculated by the model from time $t = 1$ to $t = k$, using $\beta = \hat{\beta}$ in a time window of length $2d + 1$ centered in $t = k$.

Equation 7

The estimated values of the daily disease transmission rate $\beta^*(t)$ are given by:

$\beta^*(t) = \arg\min_{\beta} J(\beta, t)$

subject to: {equation 1, equation 2

References

1. Li R. Substantial undocumented infection facilitates the rapid dissemination of novel coronavirus (SARS-CoV2). Science. 2020.

2. Degue KH, Ny JL. Estimation and outbreak detection with interval observers for uncertain discrete-time SEIR epidemic models. Int J Control. 2019;93: 2707–18. https://doi.org/10.1080/00207179.2019.1643492

3. Iggidr A, Souza MO. State estimators for some epidemiological systems. J Math Biol. 2019;78:225–56. PubMed https://doi.org/10.1007/s00285-018-1273-3

Appendix Table. SARS-CoV-2 IgG serology results and seroprevalence among blood donors from 7 cities, Minas Gerais, Brazil, 2020*

	City																				
	Pouso Alegre			Uberaba			Juiz de Fora			Belo Horizonte			Montes Claros			Governador Valadares			Uberlândia		
Mo.	–	+	%	–	+	%	–	+	%	–	+	%	–	+	%	–	+	%	–	+	%
Mar	44	0	0	44	0	0	44	0	0	44	0	0	44	0	0	44	0	0	43	1	2.3
Apr	44	0	0	42	2	4.6	44	0	0	44	0	0	44	0	0	44	0	0	44	0	0
May	58	1	1.7	60	0	0	59	1	1.7	59	1	1.7	59	1	1.7	60	0	0	60	0	0
Jun	65	0	0	64	1	1.5	64	0	0	156	2	1.3	50	0	0	63	2	3.1	70	0	0
Jul	67	3	4.3	67	3	4.3	68	2	2.9	186	5	2.6	50	0	0	66	4	5.7	67	2	2.9
Aug	76	1	1.3	51	1	1.9	83	4	4.6	189	12	6.0	60	2	3.2	97	4	4.0	64	2	3.0
Sep	66	3	4.4	66	4	5.7	76	1	1.3	204	8	3.8	47	4	7.8	77	12	13.5	136	12	8.1
Oct	95	3	3.1	99	3	2.9	111	4	3.5	157	9	5.4	53	8	13.1	126	11	8.0	219	30	12.1
Nov	136	4	2.9	138	6	4.2	160	9	5.3	377	36	8.7	62	9	12.7	192	18	8.6	324	38	10.5
Dec	188	7	3.6	196	17	8.0	222	21	8.6	369	35	8.7	103	12	10.4	274	30	9.9	372	30	7.5
Total	839	22	2.6	827	37	4.3	931	42	4.3	1,785	108	5.7	572	36	5.9	1,043	81	7.2	1,399	115	7.6

*SARS-CoV-2, severe acute respiratory syndrome coronavirus 2; –, negative; +, positive.
